# Supplementary material for: Mechanical metamaterials made of freestanding quasi-BCC nanolattices of gold and copper with ultra-high energy absorption capacity
Source: Nat Commun. 2023 Mar 4;14:1243. doi: 10.1038/s41467-023-36965-4 (PMC9985601; doi:10.1038/s41467-023-36965-4)
Supplement: Supplementary file 1 — Supplementary Information [file 41467_2023_36965_MOESM1_ESM.pdf]

Supplementary Information

Mechanical metamaterials made of freestanding quasi-BCC nanolattices of gold and copper with ultra-high energy absorption capacity

Hongwei Cheng[1,2], Xiaoxia Zhu[1,2], Xiaowei Cheng[3], Pengzhan Cai[3], Jie Liu[1,2], Huijun Yao[1,2], Ling Zhang[3]*, Jinglai Duan[1,2,4]*

[1] *Institute of Modern Physics, Chinese Academy of Sciences, Lanzhou 730000, China*

[2] *School of Nuclear Science and Technology, University of Chinese Academy of Sciences, Beijing 100049, China*

[3] *International Joint Laboratory for Light Alloys (MOE), College of Materials Science and Engineering, Chongqing University, Chongqing 400045, China*

[4] *Advanced Energy Science and Technology Guangdong Laboratory, Huizhou 516000, China*

*Correspondence to: zhangling2014@cqu.edu.cn;

j.duan@impcas.ac.cn

Supplementary Information Table of Contents

Supplementary Discussions

Supplementary Discussion 1 The microstructure and the purity of the gold and copper quasi-BCC nanolattices.

Supplementary Discussion 2 Factors affecting the mechanical properties of the gold and copper quasi-BCC nanolattices.

Supplementary Discussion 3 Theoretical calculation of the mechanical properties of the gold quasi-BCC nanolattices.

Supplementary Discussion 4 Lower limit of the relative density of the gold and copper quasi-BCC nanolattices.

Supplementary Figures

Supplementary Fig. 1 XRD data of gold and copper quasi-BCC nanolattices.

Supplementary Fig. 2 High-resolution TEM images of two interconnected gold beams.

Supplementary Fig. 3 SEM images of gold and copper quasi-BCC nanolattices.

Supplementary Fig. 4 SEM images and EDS data of gold and copper quasi-BCC nanolattices.

Supplementary Fig. 5 EELS spectra of gold and copper quasi-BCC nanolattices.

Supplementary Fig. 6 Finite element simulations of the mechanical properties of gold quasi-BCC nanolattices and periodic BCC nanolattices under same beam dimeter and relative density.

Supplementary Fig. 7 Snapshots of finite element simulations describing the deformation behavior of the in situ compression process of gold quasi-BCC nanolattices.

Supplementary Fig. 8 SEM image of a FIB-milled micropillar.

Supplementary Fig. 9 TEM image of two interconnected gold beams.

Supplementary Fig. 10 Schematic diagrams of surface contact states of gold quasi-BCC nanolattices.

Supplementary Fig. 11 Stress-strain curves of the samples Au-34 and Au-117.

Supplementary Fig. 12 Geometric models and finite element simulations of gold quasi-BCC nanolattices with ultralow relative densities.

Supplementary Fig. 13 SEM images of a gold quasi-BCC nanolattice with a relative density of 0.15.

Supplementary Tables

Supplementary Table 1 Numerical results of experimentally tested quasi-BCC nanolattices.

Supplementary Table 2 Mechanical parameters of gold materials used in finite element simulation.

Supplementary Table 3 Numerical results of finite element simulations of BCC nanolattices.

Supplementary Table 4 Compressive strength of experiments and theoretical calculations from formula (4) of gold quasi-BCC nanolattices.

Supplementary References

Supplementary Discussions

Supplementary Discussion 1 The microstructure and the purity of the gold and copper quasi-BCC nanolattices

To examine the microstructure and the purity of our gold and copper quasi-BCC nanolattices, we have performed all the following characterizations, i.e., X-ray diffraction (XRD), high-resolution TEM, backscattered electron SEM, energy disperse x-ray spectra (EDS), and electron energy loss spectroscopy (EELS). Benefiting from the above methods, we have determined that microstructures of our gold and copper quasi-BCC nanolattices are polycrystalline and, within the detection limit of the above techniques, the nanolattices are in high purity and no impurity was detected. Details are illustrated below.

The microstructures of the gold and the copper quasi-BCC nanolattices were evaluated by XRD and TEM. XRD data of either gold or copper quasi-BCC nanolattices exhibit typical polycrystalline-like patterns of face-centered cubic (FCC) phases, where the peaks of main crystal planes appear and, in each pattern, the (111) crystal plane shows the strongest diffraction intensity (Supplementary Fig. 1). We have also verified the polycrystalline microstructure by TEM, taking gold as an example (Supplementary Fig. 2). It is seen that two beams are composed of three grains, confirming the polycrystalline microstructure.

The purity of the gold and the copper were examined by backscattered electron SEM (BSE-SEM), EDS, and EELS techniques. The BSE-SEM images of gold and copper quasi-BCC nanolattices have similar morphologies as those of the secondary electron SEM (SE-SEM) images (Supplementary Fig. 3). Although the BSE-SEM images have lower signal-to-noise ratio, there is no observable contrast difference, reflecting the nanolattices have high purities at the microscale. This observation is further

supported by the EDS analysis (Supplementary Fig. 4). The EDS data taken from different regions illustrate that our nanolattices are only composed of pure gold or copper, respectively. The high purity of gold and copper quasi-BCC nanolattices are further consolidated by EELS spectra (Supplementary Fig. 5), where no other elements were detected. Based on the above results, it is safe to conclude that our nanolattices are in high purity.

Supplementary Discussion 2 Factors affecting the mechanical properties of the gold and copper quasi-BCC nanolattices

<u>Effect of offset nodes on mechanical properties of gold and copper quasi-BCC nanolattices</u>

In this work, the influence of offset nodes in the quasi-BCC nanolattice on its mechanical properties was studied through finite element simulation. Combined with the previous mechanical test results of gold pillars, gold nanowires, and nanoporous gold[1-7], the mechanical parameters of gold materials with size effects were set as shown in Supplementary Table 2. We have evaluated quasi-BCC nanolattices and periodic BCC nanolattices with the same relative densities and beam diameters (Supplementary Fig. 6, 7 & Supplementary Table 3). In our case, the influence of node offset effects on the stiffness depends on the relative density. At the relative density of 0.48, the quasi-BCC nanolattice has an 8% decrease, i.e., from 9474.2 MPa for the periodic nanolattice to 8730.5 MPa for the quasi-BCC nanolattice. At the relative density of 0.20, the quasi-BCC nanolattice has a 57% decrease, i.e., from 1259.5 MPa for the periodic nanolattice to 541.0 MPa for the quasi-BCC nanolattice. For the strength, at the relative density of 0.48, the quasi-BCC nanolattice has a 47% decrease, i.e., from 126.1 MPa for the periodic nanolattice to 66.4 MPa for the quasi-BCC nanolattice. At the relative density of 0.20, the quasi-BCC nanolattice has a 53% decrease, i.e., from 26.9 MPa for

the periodic nanolattice to 12.7 MPa for the quasi-BCC nanolattice. To sum up, the node offset effects have a greater impact on the mechanical properties of our quasi-BCC nanolattices, as comparing with those on the reported octet-truss nanolattices (Ref. 21).

Effects of surface roughness on mechanical properties of gold and copper quasi-BCC nanolattices

Surface roughness, beam waviness, misalignment of nodes, and others are factors that influence the measured stiffness of a metamaterial. In our quasi-BCC nanolattices, from the SEM image of a FIB-milled pillar (Supplementary Fig. 8), it is clearly seen that some neighboring protrusions (beam ends) are in different heights and, as a result, form surface roughness. It would be great to give a relative degree of the surface roughness. Unfortunately, unlike the surface roughness of a nonporous solid material which can be quantitatively evaluated by techniques such as atomic force microscopy, the relative degree of the surface roughness of porous materials, in particular stochastic truss porous materials like ours, are hardly evaluated quantitatively. For the beam waviness, we determined the waviness from a TEM image (Supplementary Fig. 9). The results show that the beam waviness of beam 1 is 0.5 nm and the beam waviness of beam 2 is 0.6 nm, respectively, both are below 1 nm and less than 1% (beam diameter 69±2 nm). Thus, we think beam waviness should play a minor role in influencing the stiffness, as comparing with the surface roughness.

To illustrate the effects of contact state and surface roughness on the stiffness and the strength, we have performed additional finite element simulations (Supplementary Fig. 10). The areal density and the beam diameter of the simulated quasi-BCC nanolattices are $7.1\times10^{8}\times4$ cm^{-2} and 69 nm, respectively, corresponding to those of the sample Au-69. The model volume is $1\times1\times1$ μm^{3}. Surface contact state 1 represents the highest contact level (contact area 190249 nm^{2}), namely, the upper ends of beams are fully in contact with the indenter, which is the case involved in this paper. The state 2

(contact area 98358 nm^2) and the state 3 (contact area 34620 nm^2) represent that the upper ends partly contact with the indenter with reduced contact level. In the state 4, about a half of the number of beams are in contact with the indenter at the contact level prescribed in the state 3, and the rest do not contact. Compared to the state 3, the state 4 has surface roughness. One can see that the compressive stiffness degrades from 541.0 MPa to 332.4 MPa, as the contact changes from the state 1 to the state 3 (Supplementary Fig. 10e). In comparison, the strength degrades from 12.7 MPa to 12.4 MPa, reflecting that the strength is insensitive to the contact state (Supplementary Fig. 10f). In short, the surface roughness has an obvious impact on the stiffness and a limited influence on the strength.

Complementary to the above simulations, the experimental stress-strain curve may also give some hints about the surface roughness. We found the stress-strain curves of all the samples will go through two parts at the initial stage during compression. The first part has a smaller slope E_1 and the second part has a larger slope E_2. For the Au-34 sample, the turning point locates at the strain of 0.02. The E_1 and E_2 are 851.5 MPa and 1651.5 MPa, respectively (Supplementary Fig. 11a). For the Au-117 sample, the turning point locates at the strain of 0.05. The E_1 and E_2 are 464.2 MPa and 700.3 MPa, respectively (Supplementary Fig. 11b). We think the smaller slope (E_1) of the first part is very likely due to the surface roughness and the larger slope (E_2) is the stiffness of our quasi-BCC nanolattices. This explanation may be supported by the findings reported previously[8,9]. As such, the turning point of strain reflects, to some extent, the surface roughness.

In short, both the contact level and the surface roughness have obvious impact on the stiffness and limited influence on the strength.

Supplementary Discussion 3 Theoretical calculation of the mechanical properties of the gold

quasi-BCC nanolattices

To quantitatively evaluate the effect of nodal offsets on nanolattice mechanical properties, we reviewed theoretical models for ideal cell compressive modulus and strength, whose equivalent compressive modulus for body-centered cubic (BCC) structures are[10, 11]:

$$E_{\mathrm{BCC}} = \overline{\rho} \cdot E \cdot \sin^4 \theta \tag{1}$$

where E is the Young's modulus of the parent material. For a large relative density of nanolattices (~0.5), the effective stiffness can be well reproduced by Equation 1. When the relative density is small (<0.3), the ratio of stiffness to $\overline{\rho}$ is 2.2 instead of a power of 1.0, as predicted by Ashby. This may be related to the nanolattice geometry edge effects in the study (Ref. 19), for which comparable power-law exponents have been obtained for similar nanolattices.

The compressive strength of the BCC structure can be expressed as[10, 11]:

$$\sigma_{\mathrm{BCC}} = \overline{\rho} \cdot \sin^2 \theta \cdot \min\{\sigma_y, \sigma_{\mathrm{cr}}\} \tag{2}$$

where σ_y is the yield strength of the parent material, and σ_{cr} is the ultimate strength of the bracing in the buckling state, which can be expressed as[10,11]:

$$\sigma_{\mathrm{cr}} = \frac{k^2\pi^2 I E_t}{A l^2} = \frac{k^2\pi^2 d^2 E_t}{16 l^2} \tag{3}$$

where the factor k=1 is determined by the zero rotational stiffness of the end nodes of the nanobeams, I is the moment inertia of the beam (=$\pi d^4/64$), and A is the cross sectional area of the circular beam ($\pi d^2/4$). In above equation, E_t denotes the tangent modulus of the parent material, which depends on the strain hardening characteristics, typically one-tenth of the Young's modulus. At higher relative densities (~0.5), the compressive strength of the nanolattice is governed by the yield strength σ_y of the material. However, as the relative density decreases (<0.3), the structs become more and more slender, and under certain conditions the compressive strength of the nanolattice is governed by

buckling rather than yielding. The compressive strength in this case is obtained by replacing σ_{cr} in equation 2 with the buckling strength of the truss member. In this way, we obtained the quantitative value of the compressive strength of periodic body-centered cubic structure through finite element simulation and theoretical calculation, and the two are highly consistent, the relative error is less than 5%. For the metallic quasi-BCC nanolattices in this paper, when the feature size and relative density are fixed, it is only different from the periodic body-centered cubic structure in structure. Or we can consider quasi-BCC nanolattices as nanolattice structures with full-node offset defects. Its compressive strength could be given by:

$$\sigma_{\mathrm{BCC}} = \eta \cdot \overline{\rho} \cdot \sin^2 \theta \cdot \min\{\sigma_y, \sigma_{\mathrm{cr}}\} \quad (4)$$

Where η is the structure factor, which is numerically equal to 0.5, and we can accurately calculate the compressive strength of the quasi-BCC nanolattice by Equation (4), see details in Supplementary Table 4. The high agreement between the above theoretical calculation results and the experimental results of gold and copper quasi-BCC nanolattice shows the generality of the theory.

Supplementary Discussion 4 Lower limit of the relative density of the gold and copper quasi-BCC nanolattices

For a single monolithic quasi-BCC nanolattice, the lower density limit is basically determined by the connectivity of beams and, in turn, by the areal density, the diameter, and the length of nanobeams (a longer beam has more possibilities to connect to other beams). To elaborate on the lower limit of the relative density, we have carried out the analysis with the aid of geometric models and finite element simulations (Supplementary Fig. 12) in combination with experimental test (Supplementary Fig. 13), using gold as the material. The analysis of geometric models and finite element simulations

suggest that, the quasi-BCC nanolattice of the relative density of only 0.01 nearly remains structural integrity, given that it is in the same dimensions of FIB milled pillars, i.e., 10×10×10 μm^3 (Supplementary Fig. 12). However, because of the morphological damage induced by the surface tension of dichloromethane during dissolving polycarbonate template, the experimental test shows that the quasi-BCC nanolattice with a relative density of 0.15 starts to lose its structural integrity partially (Supplementary Fig. 13). Details are elucidated below.

The analyses of geometric models and finite element simulations were carried out to estimate the lower limit of relative density from the perspective of structural integrity and performance degradation, respectively. For the analyses, the areal density was fixed to be $7.1\times10^8\times4$ cm^{-2}, corresponding to that of the sample Au-117. At this fixed areal density, the relative densities of 0.15, 0.1, 0.05, and 0.01 were selected to analyze the connectivity of beams by choosing beam diameters of 60 nm, 50 nm, 34 nm, and 15 nm, respectively. From the geometric models of relative density of 0.01 (Supplementary Fig. 12a-c), we found that the connectivity of beams increases with enlarging model volume of nanolattice. In the case of model volume of 1×1×1 μm^3, 25% beams fail to connect to any other beams, namely, the nanolattice loses its partial structural integrity (Supplementary Fig. 12a). The percentage becomes to 8% as the model volume increases to 2×2×2 μm^3 and further reaches 5% for the model volume of 3×3×3 μm^3. Although we could not enlarge the model volume further due to limited computational resources, it is reasonable to speculate that, at the volume of 10×10×10 μm^3 which is the case for our real mechanical tests, nearly 100% beams would connect to other beams. Namely, the nanolattices are monolithic and have their structural integrity, despite the relative density down to 0.01.

In addition to the analysis based on geometric model, we further simulated the mechanical responses of the quasi-BCC nanolattices with low relative densities. In simulations, the areal density

of beams was fixed to be $7.1\times10^8\times4$ cm^{-2}, corresponding to that of the sample Au-117. The relative density of the gold quasi-BCC nanolattice decreases by reducing the beam diameter. The relative densities of 0.15, 0.1, 0.05, and 0.01 were tested by choosing beam diameters of 60 nm, 50 nm, 34 nm, and 15 nm, respectively. The nanolattice thickness was chosen to be 1 μm. In finite element simulations, those beams unconnected to any other beam were manually removed and did not contribute to mechanical responses. The simulated stress-strain curves show that all the nanolattices have successful mechanical responses (Supplementary Fig. 12d). Moreover, it is seen that the stiffness and the strength are highly dependent on the relative density (Supplementary Fig. 12e,f). In summary, the relative density as low as 0.01 nearly keeps the structural integrity and yields certain mechanical strength.

We have also searched the lower limit of the relative density under our experimental conditions. We found that the gold quasi-BCC nanolattice with a relative density of 0.15 starts to lose its structural integrity partially, which is attributed to the morphological damage induced by the surface tension of dichloromethane solvent during dissolving polycarbonate template. For this sample, the area density is $7.1\times10^8\times4$ cm^{-2} (consistent with the sample Au-117), and the beam diameter is 60±3 nm. The SEM images of the quasi-BCC nanolattices are shown in Supplementary Fig. 13. It is seen that, although the sample keeps monolithic (Supplementary Fig. 13a), the surface morphology is partially damaged at the microscopic scale (Supplementary Fig. 13b). Guided by the analyses of geometric models and finite element simulations, it is reasonable to anticipate the quasi-BCC nanolattices with lower relative densities below 0.2 could be experimentally fabricated by further refining the experimental process, for example, reducing or eliminating surface tension of solvents using a freeze-drying method[12].

Supplementary Figures

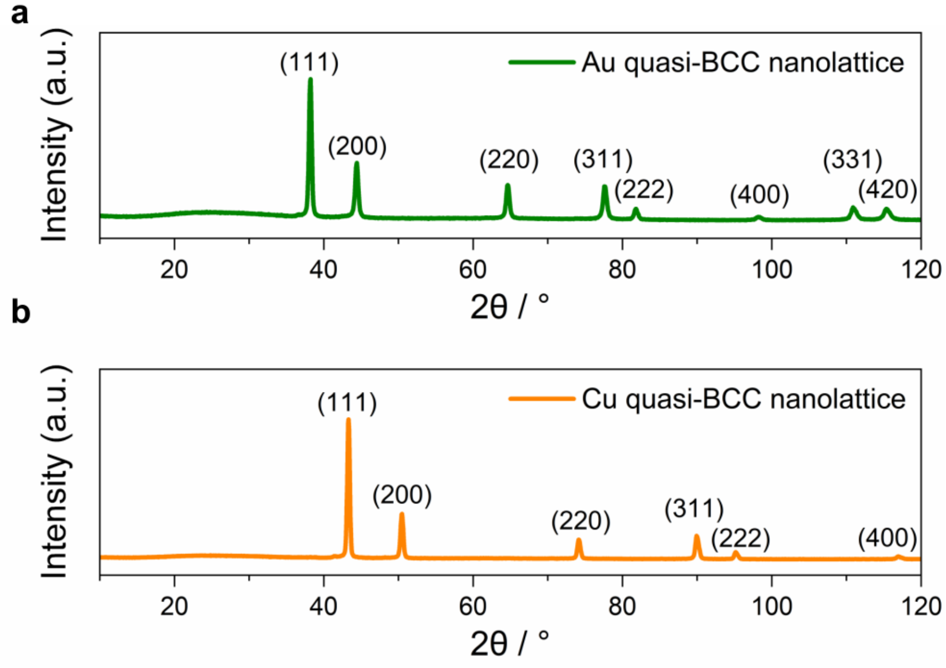

Supplementary Fig. 1 XRD data of gold and copper quasi-BCC nanolattices. a Gold. **b** Copper. Source data are provided as a Source Data file.

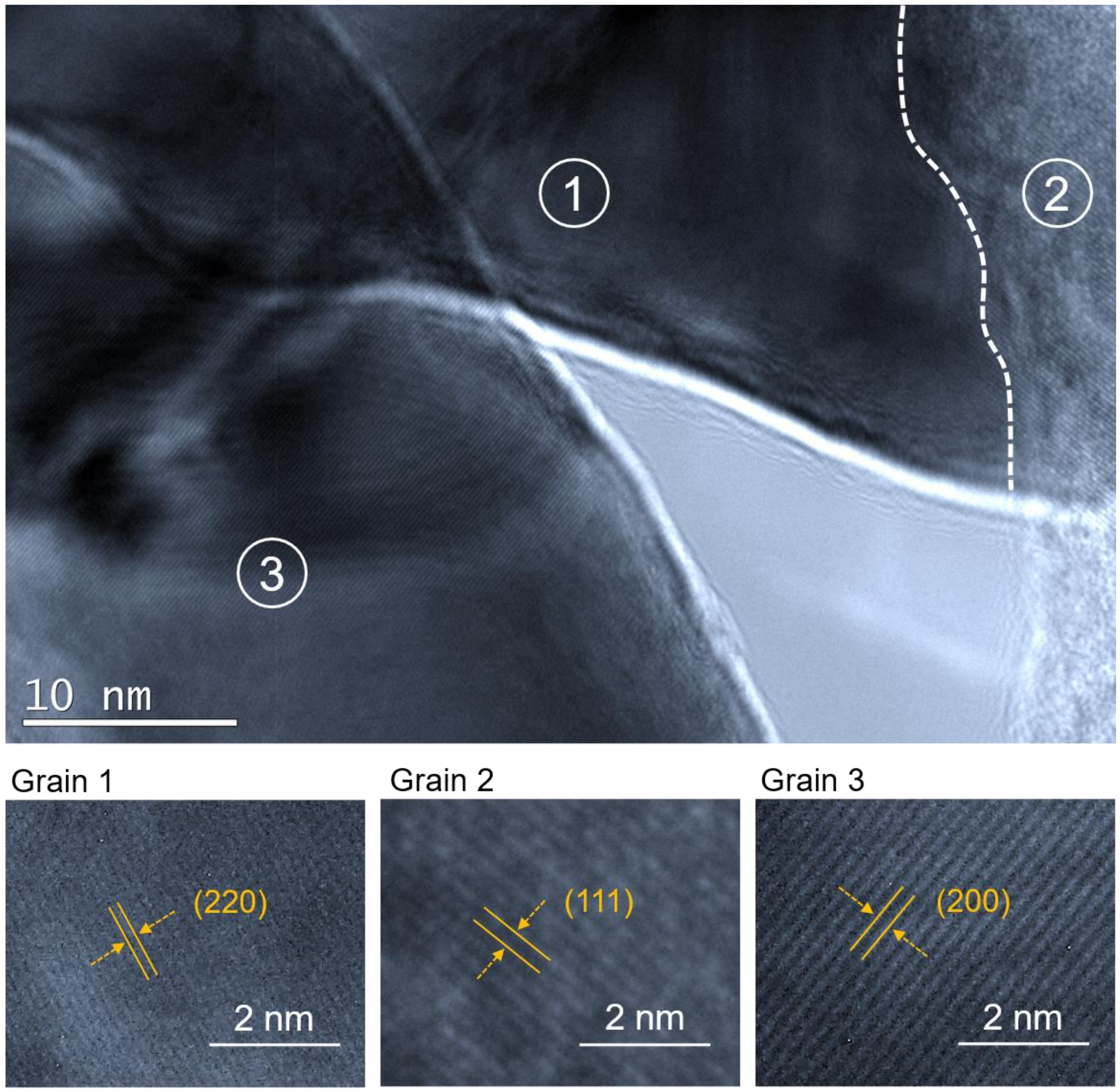

Supplementary Fig. 2 High-resolution TEM images of two interconnected gold beams.

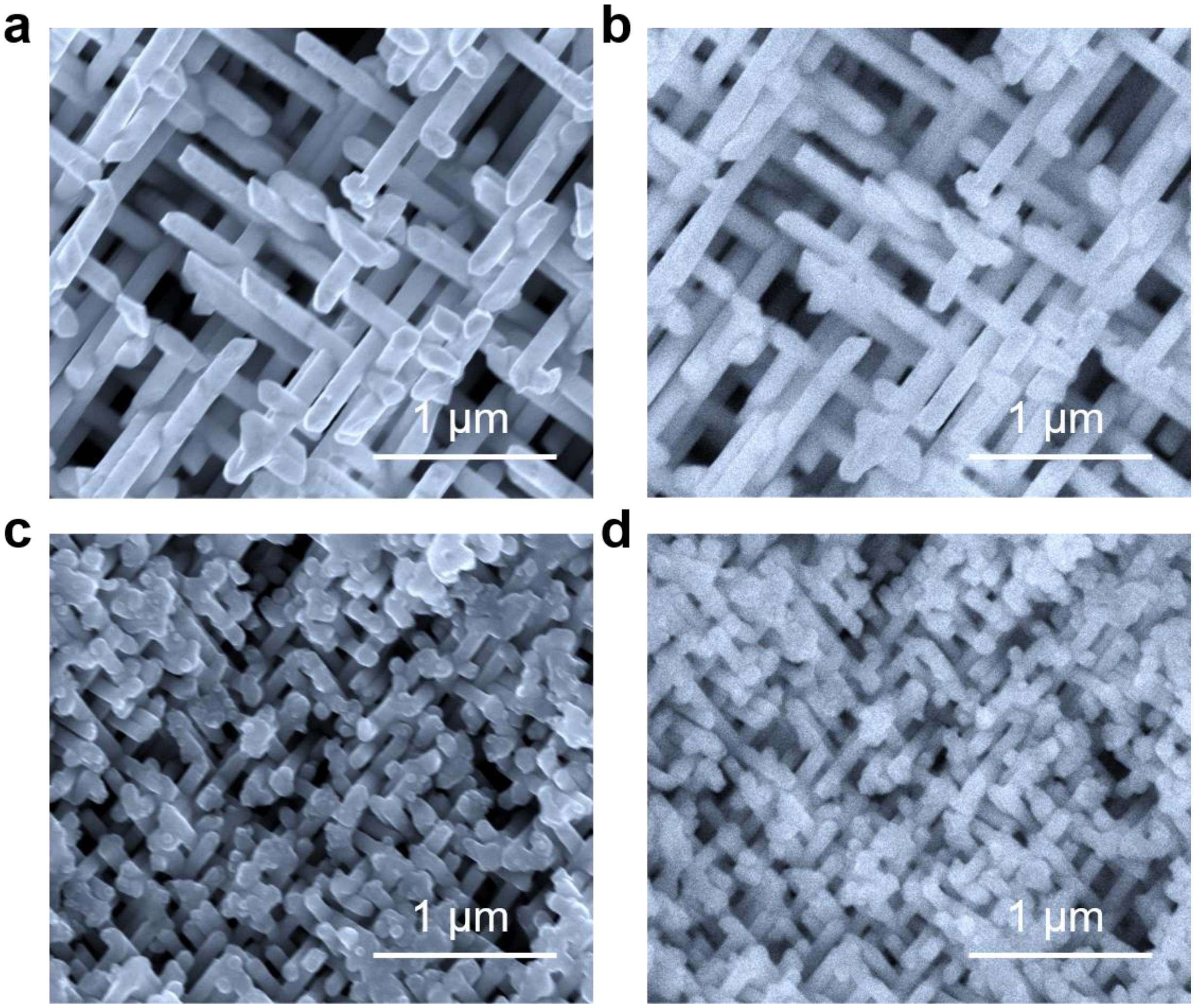

Supplementary Fig. 3 SEM images of gold and copper quasi-BCC nanolattices. a Secondary electron SEM image of a gold quasi-BCC nanolattice. **b** Backscattered electron SEM image taken from the same region shown in **a**. **c** Secondary electron SEM image of a copper quasi-BCC nanolattice. **d** Backscattered electron SEM image taken from the same region shown in **c**. The areal density and the beam diameter for the gold quasi-BCC nanolattice are $3.5\times10^{8}\times4$ cm^{-2} and 131±4 nm, respectively. The areal density and the beam diameter for the copper quasi-BCC nanolattice are $2.1\times10^{9}\times4$ cm^{-2} and 86±4 nm, respectively.

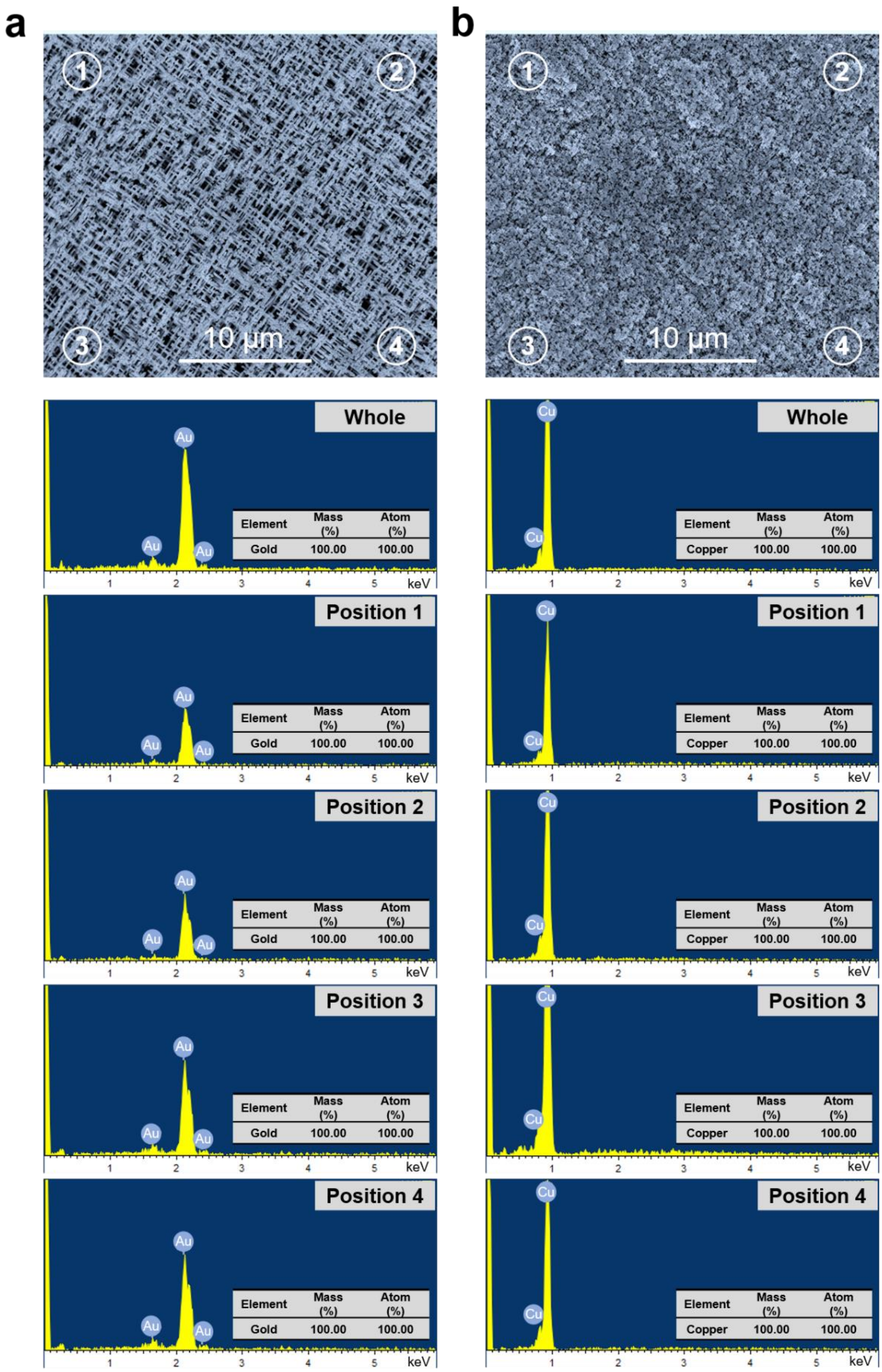

Supplementary Fig. 4 SEM images and EDS data of gold and copper quasi-BCC nanolattices. a SEM image and corresponding EDS spectra of a gold quasi-BCC nanolattice. **b** SEM image and corresponding EDS spectra of a copper quasi-BCC nanolattice.

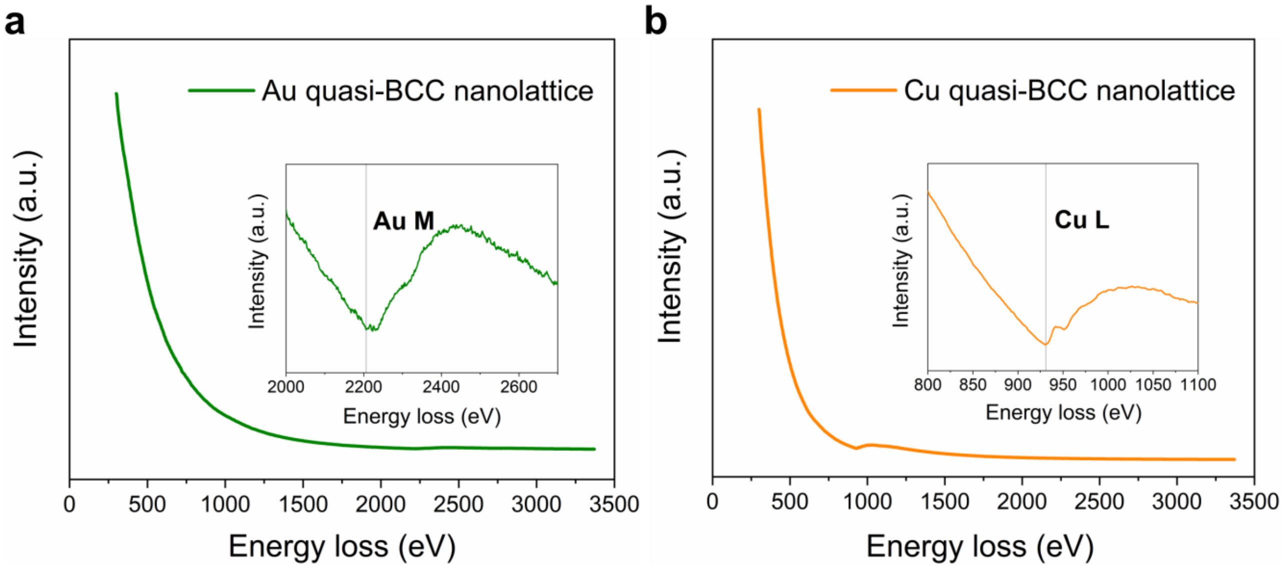

Supplementary Fig. 5 EELS spectra of gold and copper quasi-BCC nanolattices. a Gold quasi-BCC nanolattice. **b** Copper quasi-BCC nanolattice. Source data are provided as a Source Data file.

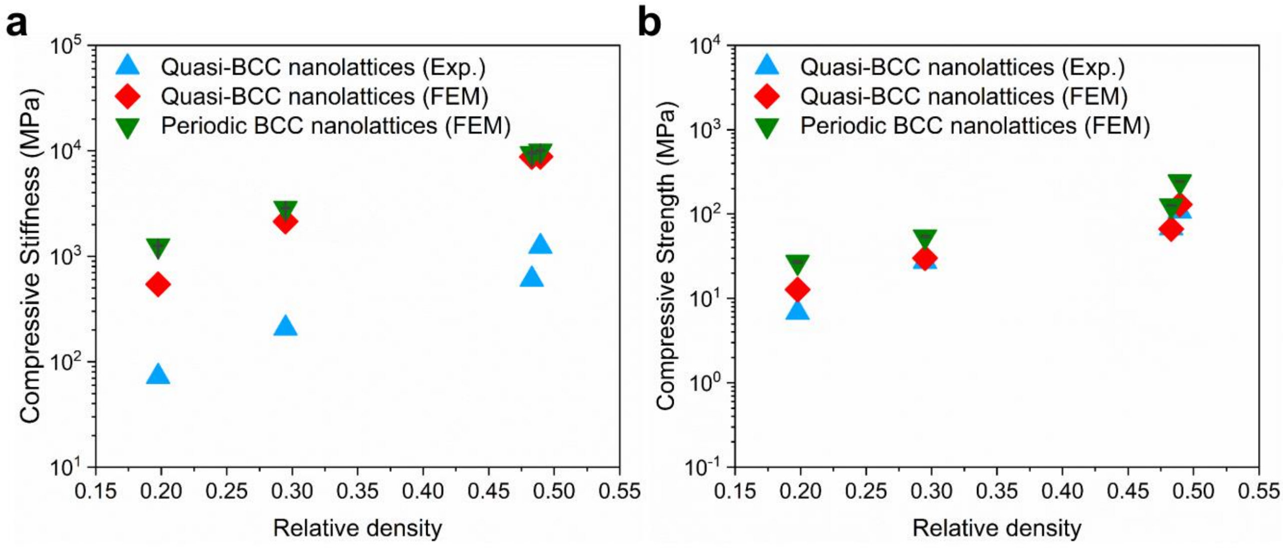

Supplementary Fig. 6 Finite element simulations of the mechanical properties of gold quasi-BCC nanolattices and periodic BCC nanolattices under same beam dimeter and relative density. a Compressive stiffness versus relative density and **b** Compressive strength versus relative density. Source data are provided as a Source Data file.

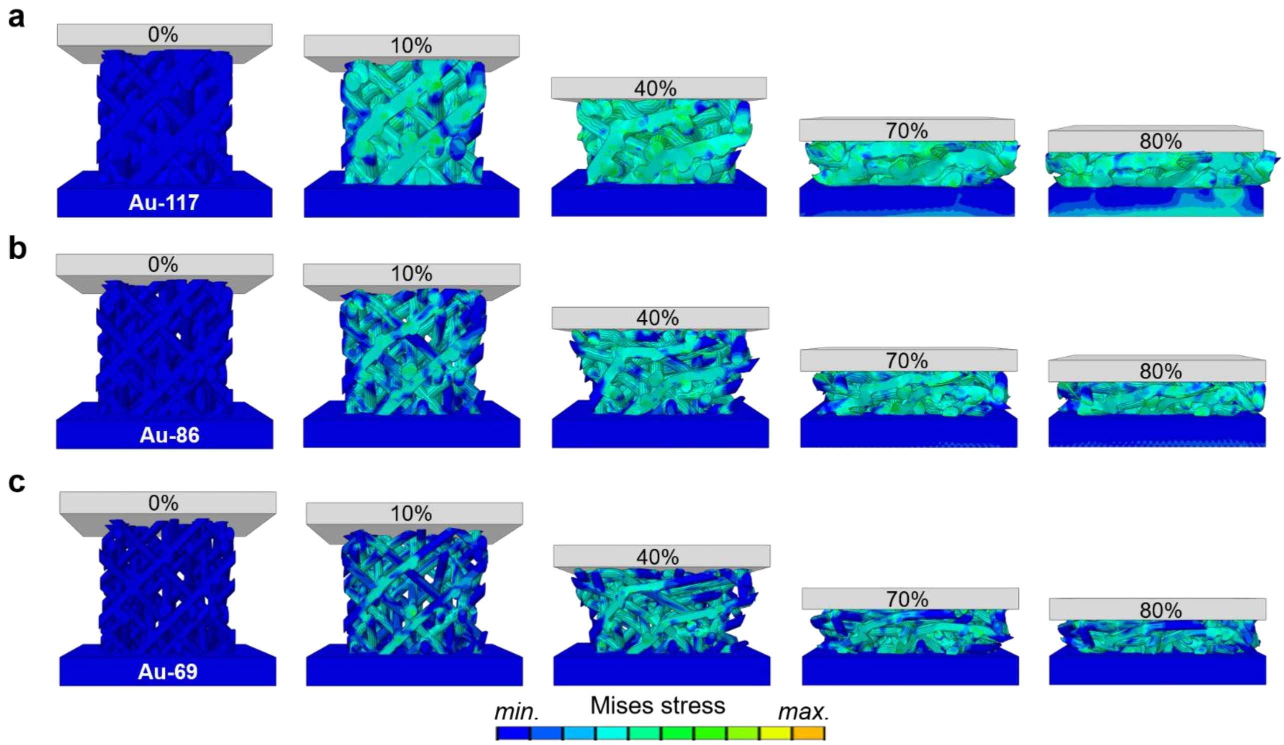

Supplementary Fig. 7 Snapshots of finite element simulations describing the deformation behavior of the in situ compression process of gold quasi-BCC nanolattices. a Au-117 sample at different compression stages. **b** Au-86 sample at different compression stages. **c** Au-69 sample at different compression stages.

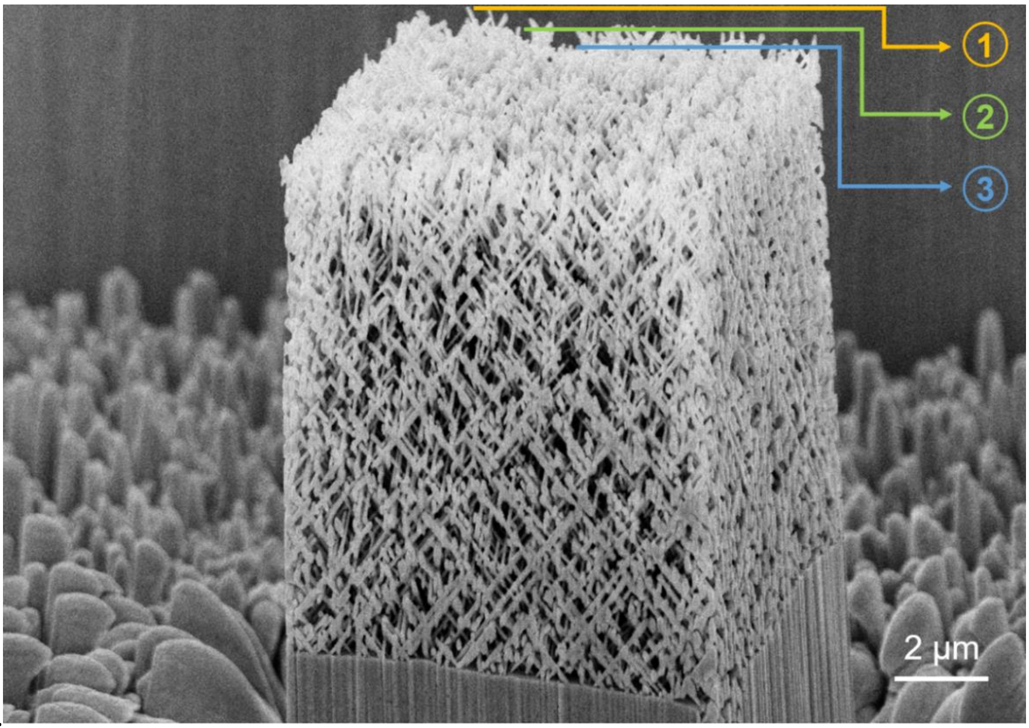

Supplementary Fig. 8 SEM image of a FIB-milled micropillar.

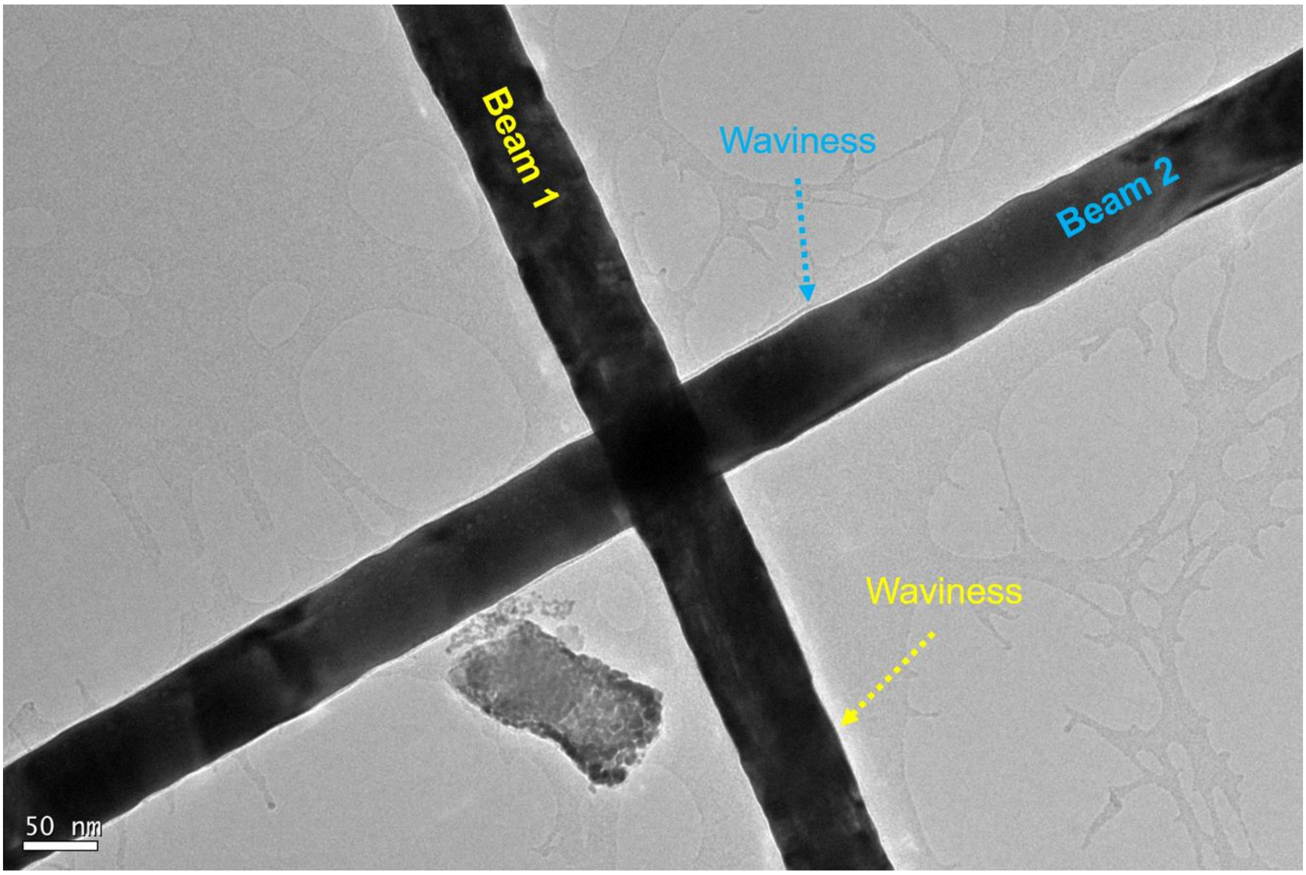

Supplementary Fig. 9 TEM image of two interconnected gold beams.

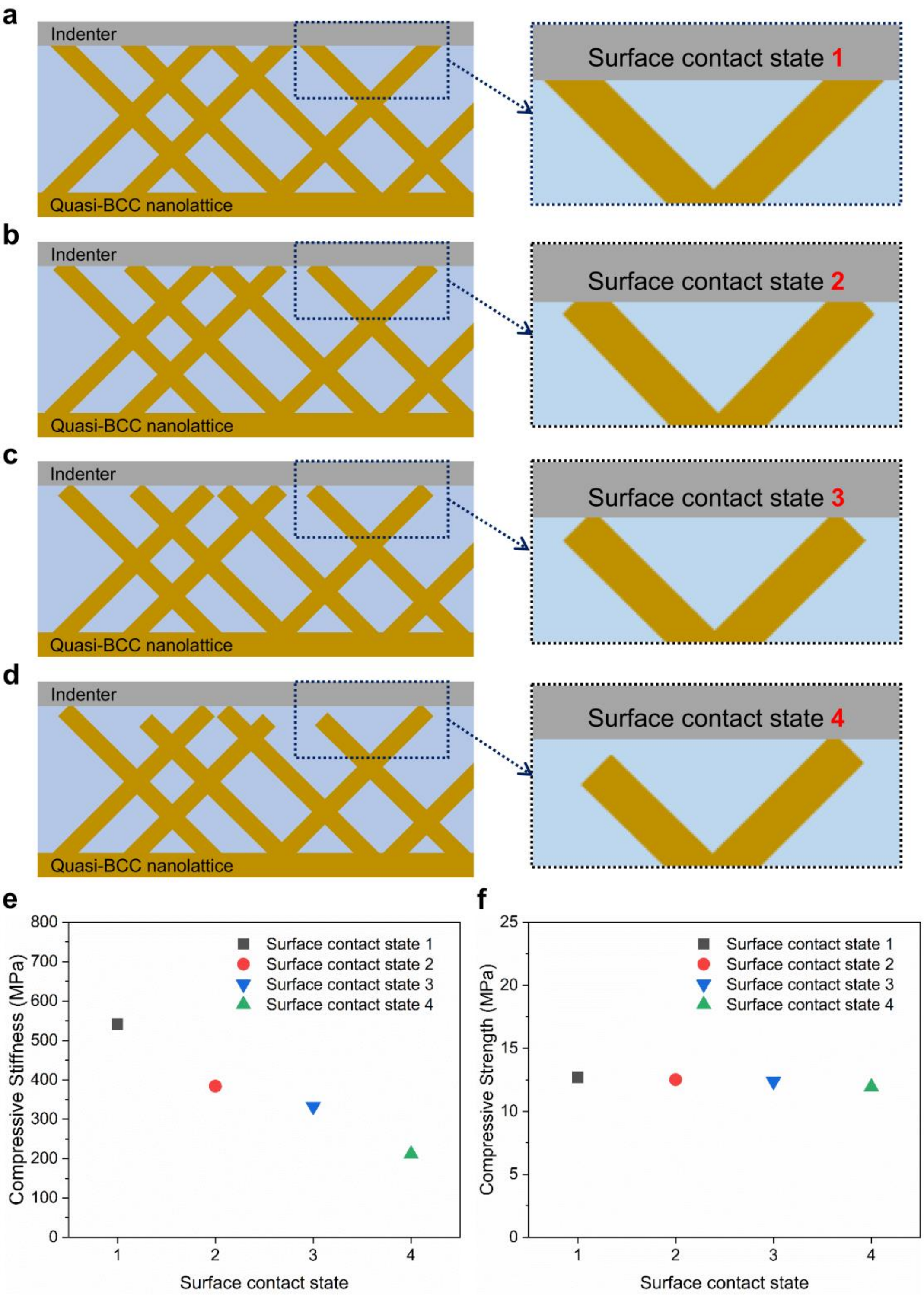

Supplementary Fig. 10 Schematic diagrams of surface contact states of gold quasi-BCC nanolattices. a Surface contact state 1, the upper end of the nanobeam is fully in contact with the indenter, with a maximum contact area 190249 nm^2, which is also the contact state used in the finite element simulation involved in this paper. **b** Surface

contact state 2, a large part of the upper end of the nanobeam is in contact with the indenter, with a contact area 98358 nm^2. **c** Surface contact state 3, a small part of the upper end of the nanobeam is in contact with the indenter, with a contact area 34620 nm^2. **d** Surface contact state 4, because of surface roughness, not all the beams are in contact with the indenter. For the beams in contact with the indenter, the contact area of each beam equals to that of the contact state 3. **e** Compression stiffness versus contact state. **f** Compression strength versus contact state. In these simulations, the areal density ($7.1\times10^8\times4$ cm^{-2}), the relative density (0.20), and the beam diameter (69 nm) are identical to those of Au-69. Source data are provided as a Source Data file.

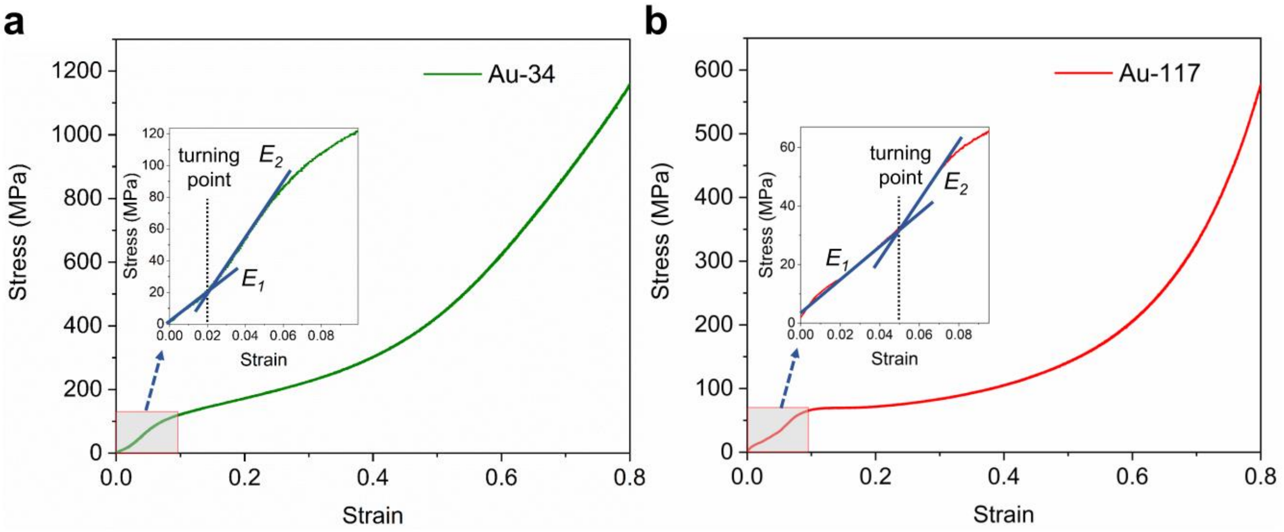

Supplementary Fig. 11 Stress-strain curves of the samples Au-34 and Au-117. a Au-34. **b** Au-117. Source data are provided as a Source Data file.

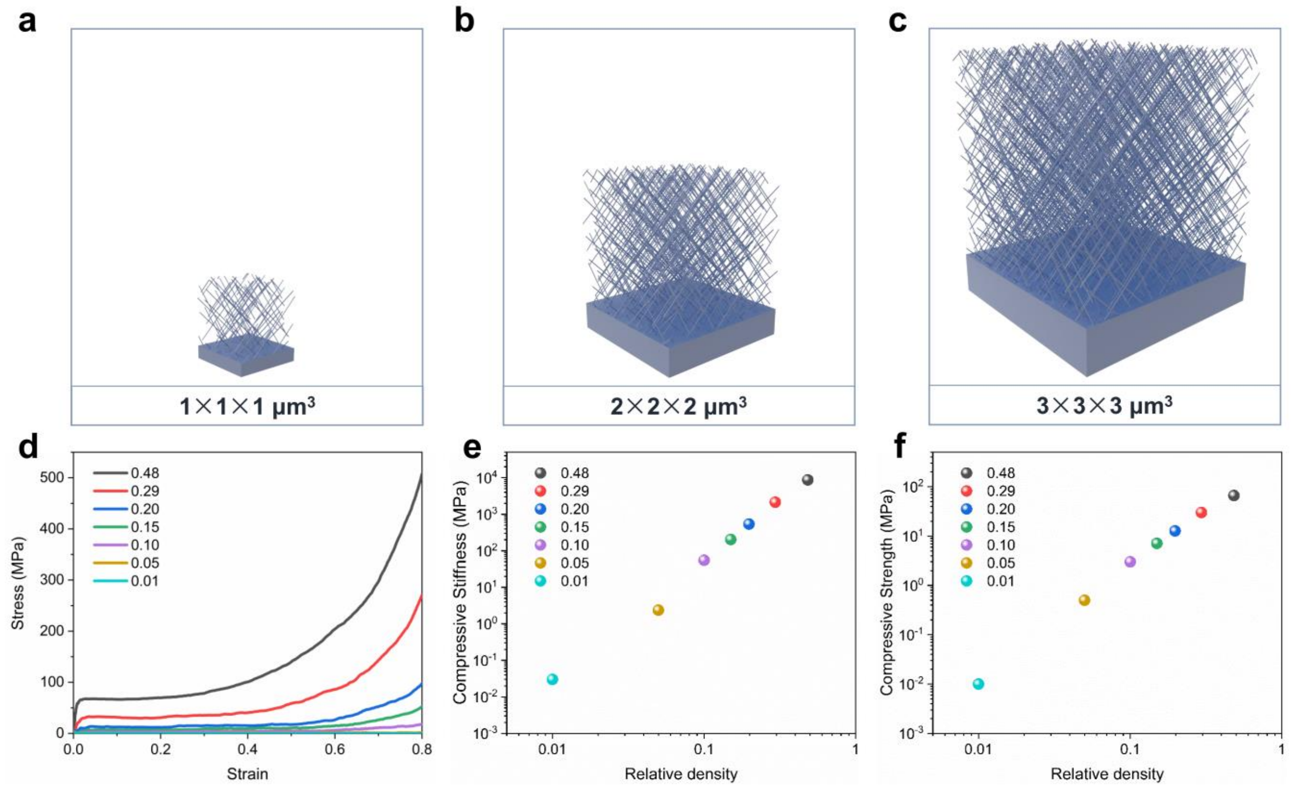

Supplementary Fig. 12 Geometric models and finite element simulations of gold quasi-BCC nanolattices with ultralow relative densities. a Model volume 1×1×1 μm^3. **b** Model volume 2×2×2 μm^3. **c** Model volume 3×3×3 μm^3. **d** Simulated stress-strain curve. **e** Compressive stiffness versus relative density. **f** Compressive strength versus relative density. Source data are provided as a Source Data file.

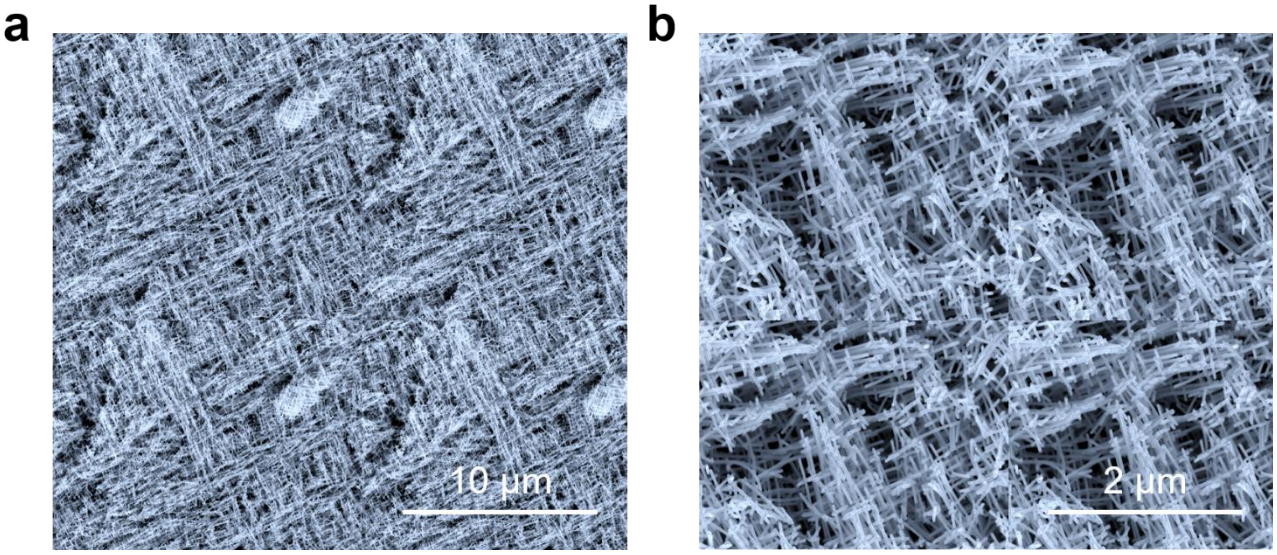

Supplementary Fig. 13 SEM images of a gold quasi-BCC nanolattice with a relative density of 0.15. a Low-magnification. **b** Magnified image of **a**.

Supplementary Tables

Supplementary Table 1 Numerical results of experimentally tested quasi-BCC nanolattices. Mean values and corresponding standard deviations are based on at least three measurements.

Sample	Stiffness (MPa)	Yield strength (MPa)	Densification strain	Energy absorption capacity (MJ m^{-3})
Au-117	604.3±86.3	68.0±1.2	0.51±0.02	57.3±5.5
Au-86	207.4±43.6	27.3±3.5	0.61±0.01	19.3±2.8
Au-69	72.1±12.7	6.8±1.1	0.65±0.01	13.0±2.3
Au-34	1234.9±70.6	107.0±11.5	0.48±0.03	100.3±6.4
Cu-34	1596.3±194.6	153.3±15.3	0.47±0.03	110.1±9.8

Supplementary Table 2 Mechanical parameters of gold materials used in finite element simulations.

Quasi-BCC nanolattice	Young's modulus (MPa)	Poisson's ratio	Strength (MPa)
Au-117	78000	0.42	550
Au-86			645
Au-69			700
Au-34			1000

Supplementary Table 3 Numerical results of finite element simulations of BCC nanolattices.

Relative density & Beam diameter	Quasi-/Periodic	Stiffness (MPa)	Yield strength (MPa)
0.48, 117 nm (Au-117)	Quasi-BCC	8730.5	66.4
	Periodic BCC	9474.2	126.1
0.29, 86 nm (Au-86)	Quasi-BCC	2140.3	30.1
	Periodic BCC	2865.2	54.0
0.20, 69 nm (Au-69)	Quasi-BCC	541.0	12.7
	Periodic BCC	1259.5	26.9
0.49, 34 nm (Au-34)	Quasi-BCC	8783.1	129.5
	Periodic BCC	9996.4	242.2

Supplementary Table 4 Compressive strength of experiments and theoretical calculations from formula (4) of gold quasi-BCC nanolattices.

Quasi-BCC nanolattice	Au-117	Au-86	Au-69	Au-34
Experiment strength (MPa)	68.1±1.2	27.3±3.5	6.8±1.1	107.0±11.4
Theoretical strength (MPa)	66.0	29.9	12.9	122.5

Supplementary References

1. Wu B., Heidelberg A. & Boland J. J. Mechanical properties of ultrahigh-strength gold nanowires. *Nat. Mater.* **4**, 525-529 (2005).
2. Dou R. & Derby B. The strength of gold nanowire forests. *Scr. Mater.* **59**, 151-154 (2008).
3. Greer J. R., Oliver W. C. & Nix W. D. Size dependence of mechanical properties of gold at the micron scale in the absence of strain gradients. *Acta Mater.* **53**, 1821-1830 (2005).
4. Volkert C. A. & Lilleodden E. T. Size effects in the deformation of sub-micron Au columns. *Philos. Mag.* **86**, 5567-5579 (2006).
5. Liu L.-Z., Ye X.-L. & Jin H.-J. Interpreting anomalous low-strength and low-stiffness of nanoporous gold: Quantification of network connectivity. *Acta Mater.* **118**, 77-87 (2016).
6. Mameka N., Wang K., Markmann J., Lilleodden E. T. & Weissmüller J. Nanoporous gold-testing macro-scale samples to probe small-scale mechanical behavior. *Mater. Res. Lett.* **4**, 27-36 (2016).
7. Weissmüller J., Newman R. C., Jin H.-J., Hodge A. M. & Kysar J. W. Nanoporous metals by alloy corrosion: formation and mechanical properties. *MRS Bull.* **34**, 577-586 (2009).
8. Maggi, A., Li, H., & Greer, J. R. Three-dimensional nano-architected scaffolds with tunable stiffness for efficient bone tissue growth. *Acta Biomater.* **63**, 294-305 (2017).
9. Gross, A., Pantidis, P., Bertoldi, K., & Gerasimidis, S. Correlation between topology and elastic properties of imperfect truss-lattice materials. *J. Mech. Phys. Solids* **124**, 577-598 (2019).
10. Wadley H. N. Multifunctional periodic cellular metals. *Philos. T. R. Soc. A* **364**, 31-68 (2006).
11. Lei H., et al. Evaluation of compressive properties of SLM-fabricated multi-layer lattice structures by experimental test and μ-CT-based finite element analysis. *Mater. Des.* **169**, 107685 (2019).
12. Gilbert, D. A., et al. Tunable low density palladium nanowire foams. *Chem. Mater.* **29**, 9814-

9818(2017).
