# Supplementary material for: Mechanical metamaterials made of freestanding quasi-BCC nanolattices of gold and copper with ultra-high energy absorption capacity
Source: Nat Commun. 2023 Mar 4;14:1243. doi: 10.1038/s41467-023-36965-4 (PMC9985601; doi:10.1038/s41467-023-36965-4)
Supplement: Supplementary file 3 — Description of Additional Supplementary Files [file 41467_2023_36965_MOESM3_ESM.pdf]

Description of Additional Supplementary Files

File Name: Supplementary Movie 1
Description: Uniaxial in situ compression testing of Au-69 quasi-BCC nanolattice.
